# Consideration of Commercially Available Hepatocytes as Cell Sources for Liver-Microphysiological Systems by Comparing Liver Characteristics

**DOI:** 10.3390/pharmaceutics15010055

**Published:** 2022-12-24

**Authors:** Shinichiro Horiuchi, Yukie Kuroda, Yuji Komizu, Seiichi Ishida

**Affiliations:** 1 Division of Pharmacology, National Institute of Health Sciences, Kawasaki 210-9501, Japan; 2 Division of Applied Life Science, Graduate School of Engineering, Sojo University, Kumamoto 860-0082, Japan

**Keywords:** microphysiological systems, liver-MPS, drug-induced liver injury, ADME, commercially available alternative hepatocyte sources, hepatocyte-like cells derived from human induced pluripotent stem cells, PXB-cells, cryopreserved human hepatocytes, cytochrome P450, liver characteristics

## Abstract

In recent years, microphysiological systems (MPS) have been developed to shorten the test period and reduce animal experiments for drug development. We examined cell sources for the liver-MPS, i.e., MPS mimicking liver function. For liver-MPS, liver-like cells with high liver functions are required. Cryo-preserved hepatocytes (cryoheps), the gold standard hepatocytes for in vitro drug development, present several disadvantages, including differences between lots due to individual donor variations or a limited cell supply from the same donor. As such, alternatives for cryoheps are sought. Hepatocyte-like cells derived from human induced pluripotent stem cells (hiPSC-Heps), hepatocytes derived from liver-humanized mice (PXB-cells), and human liver cancer cells (HepG2 cells) were examined as source candidates for liver-MPS. Gene expression levels of the major cytochrome P450 of hiPSC-Heps, PXB cells, and HepG2 cells were compared with 22 lots of cryoheps, and the activities of hiPSC-Heps were compared with 8 lots of cryopreserved hepatocytes. A focused DNA microarray was used for the global gene analysis of the liver-like characteristics of hiPSC-Heps, PXB-cells, cryoheps, and HepG2 cells. Gene expression data from the focused microarray were analyzed by principal component analysis, hierarchical clustering, and enrichment analysis. The results indicated the characteristics of individual hepatocyte cell source and raised their consideration points as an alternative cell source candidate for liver-MPS. The study contributes to the repetitive utilization of a robust in vitro hepatic assay system over long periods with stable functionality.

## 1. Introduction

Drug development is a time-consuming process (approximately 10–15 years) and costs on the order of approximately 3 billion dollars per drug. Furthermore, successfully launched drugs have the risk of market withdrawal. Drug-induced liver injury (DILI) is the major reason for withdrawing a launched drug from the market [1]. Animal testing is used in the safety evaluation of new compounds in the non-clinical testing phase. To evaluate the occurrence of side adverse events, we need to rely on animal testing. However, compared to in vitro testing, animal testing requires a long period and a large quantity of compounds, leading to higher costs and longer periods for drug development. Additionally, pharmacokinetics, which must take into account the absorption, distribution, metabolism, and excretion of compounds in the body (ADME), is known to be species-dependent and extrapolation of animal data to humans is limited. In recent years, microphysiological systems (MPS) have been actively developed to shorten the test period and reduce animal experiments for drug development [2,3,4,5,6,7,8,9,10,11,12,13,14,15]. MPS is an in vitro culture system in which culture cells in a precisely controlled environment in a microspace manufactured using MEMS (micro-electromechanical systems) technology, in order to bring out in vivo-like functions from human cells. In MPS development, the choice of the cell source to mount on MPS is as important a choice as the design of the device. For the liver-MPS, i.e., MPS mimicking the liver, liver-like cells with high liver functions such as drug-metabolizing ability and basal metabolism (glucose lipid, ammonia), are required. In drug discovery research, an assay system that can be evaluated stably for toxicity and efficacy is required [9,16]. A sustainable supply of cells with the same performance is also required for this purpose. From the above, we hypothesize that the main selection criteria for the cell source to be mounted on the liver-MPS are high liver function and a sustainable supply of cells with the same performance.

In the early stages of drug development, in vitro assay systems using cryopreserved human hepatocytes (cryoheps) are used for drug safety evaluation. However, cryoheps have some problems, which are differences between donors and a limited cell supply from the same donor. For this reason, in assay systems using cryoheps, stable evaluation is difficult because cells from the same donor cannot be used for repeated tests over a long period. In this study, we analyzed the characteristics of three commercially available types of hepatocytes that provide a long-term supply of cells from the same donor, evaluating their availability as cell sources for the liver-MPS.

As potential cell sources for cryoheps for the liver-MPS, hepatocyte-like cells derived from human induced pluripotent stem cells (hiPSC-Heps), hepatocytes derived from liver-humanized mice cells, and human liver cancer cells (HepG2 cells) were studied for the hepatocyte-like characteristic analysis. hiPSC-Heps have demonstrated hepatocyte-like phenotypes and functions, such as albumin expression, glycogen storage, urea production, bile canaliculi network formation, as well as basal expression and induction of major CYP isoforms [17,18,19,20,21,22,23,24,25,26,27,28,29,30,31,32,33,34]. PXB-cells (PhoenixBio Co. Ltd., Hiroshima, Japan) are hepatocytes derived from a PXB-mouse^®^, a chimeric mouse model with a humanized liver repopulated by transplanted human hepatocytes. PXB-cells have demonstrated human CYP enzyme [35,36], phase II enzyme [37], and transporter [38] expression and the potential for CYP enzyme induction with inducers [39,40]. HepG2 cells are derived from human hepatoma cells and have long been studied extensively at the laboratory level, including the studies for lipid and glucose metabolism [41,42] and mitochondrial toxicity [43]. However, their CYP activity is lower than that of cryoheps [44].

Although hiPSC-hep, PXB-cells, and HepG2 cells can be sustainably supplied, liver functions that are equivalent to cryoheps are required in order to utilize them for the liver-MPS. In this study, we compared the CYP metabolic abilities of hiPSC-Heps, PXB-cells, and HepG2 cells with that of cryoheps. In addition, using a focused microarray customized for liver characteristic analysis, we comprehensively compared the liver-like characteristics of these cell sources for the liver-MPS (hiPSC-hep, PXB-cells, and HepG2) with that of cryoheps. The gene expression data from the focused microarray assay were analyzed by principal component analysis (PCA), hierarchical clustering, and enrichment analysis, to compare liver-like function and characteristics among HepG2 cells, hiPSC-Heps, PXB-cells, and CryoHeps. Lastly, based on the results of the previous examinations, we evaluated the potential of HepG2 cells, hiPSC-Heps, and PXB-cells as alternatives to cryoheps and considered points for using them as cell sources for the liver-MPS. We report that the liver-like characteristics of each hepatocyte could be demonstrated using the analysis method described above and that the results raised their consideration points as an alternative cell source candidate for liver-MPS. Additionally, the possibility of mixing intestinal characteristics is raised as the consideration point for the liver-like characteristics evaluation of hepatocyte-like cells.

## 2. Materials and Methods

### 2.1. Cell Culture

Cryoheps were obtained from five vendors (Biopedric International, St Grégoire, France; Bioreclamation IVT, Baltimore, MD, USA; In Vitro ADMET Laboratories, Columbia, MD, USA; KaLy-cell, Plobsheim, France; TRL Lonza, Morrisville, NC, USA), and cultured according to the protocol recommended by the vendors. In addition, pooled cryoheps were obtained from two vendors, and cultured according to the protocol recommended by the vendors. hiPSC-Heps were obtained from three vendors (Cellular Dynamics International, Madison, WI, USA; Takara Bio Europe AB, Göteborg, Sweden; ReproCELL, Kanagawa, Japan), and cultured according to the protocol recommended by the vendors (Table S1). PXB-cells (PhoenixBio Co., Ltd., Hiroshima, Japan) are hepatocytes derived from a PXB-mouse^®^, a chimeric mouse model with a humanized liver repopulated with transplanted human hepatocytes. These cells were cultured according to the protocol recommended by the vendor. HepG2 cells are derived from a human hepatoblastoma cell line (American Type Culture Collection, Rockville, MD, USA). These cells were cultured in HyClone Dulbecco’s Modified Eagle Medium (Thermo Scientific, Rockford, IL, USA) supplemented with 10% fetal bovine serum at 37 °C in humidified air containing 5% CO_2_.

### 2.2. RNA Isolation

Cultured cells were washed twice with Dulbecco’s phosphate-buffered saline (Sigma–Aldrich, St Louis, MO, USA). Total RNAs were isolated from cells by using the RNeasy total RNA extraction kit (Qiagen, Hilden, Germany) according to the manufacturer’s instructions.

### 2.3. Measurement of Gene Expression by a TaqMan Real-Time PCR

The expression level was measured by qPCR with 8 ng of total RNA. Reverse transcription of total RNA was performed using the High Capacity RNA-to-cDNA kit (Thermo Fisher Scientific, Waltham, MA, USA) according to the manufacturer’s instructions. Gene expression levels were measured using the QuantStudio 7 Flex Real-Time PCR system (Applied Biosystems, Foster City, CA, USA). The following primers and probe sets were used to detect each gene transcript: *CYP1A2* (Hs00167927_m1), *CYP2B6* (Hs04183483_g1), *CYP2C9* (Hs00426397_m1), *CYP2C19* (Hs00426380_m1), *CYP2D6* (Hs00164385_m1), *CYP2E1* (Hs00559368_m1), *CYP3A4* (Hs00430021_m1), *CYP3A5* (Hs00241417_m1), *CYP3A7* (Hs00426361_m1), *UGT1A1* (Hs02511055_s1), *ABCB1* (Hs00184500_m1), *ABCC2* (Hs00166123_m1), *AHR* (Hs00169233_m1), *NR1I2* (Hs00243666_m1), *RXRA* (Hs00172565_m1), *HNF4* (Hs00230853_m1), *CEBPA* (Hs00269972_s1), *VIL1* (Hs01031724_m1), *SLC15A1* (Hs00192639_m1), and *ISX* (Hs01368145_m1). The expression levels of the genes are presented relative to RNAs derived from a human liver (BioChain Institute, Inc., Newark, CA, USA).

### 2.4. Measurement of Metabolic Activity of CYP Enzymes with LC-MS/MS

Cells were incubated in William’s E Medium supplemented with the Hepatocyte Maintenance Supplement Pack (CM4000; Gibco; Durham, NC, USA) containing a cocktail of CYP probe substrates (phenacetin 50 µM (for CYP1A2), diclofenac 5 µM (for CYP2C9), mephenytoin 100 µM (for CYP2C19), bufuralol 10 µM (for CYP2D6), midazolam 5 µM (for CYP3A)) at 37 °C. After 60 min incubation, the incubation media were collected and samples were kept at −80 °C until performing liquid chromatog-raphy-tandem mass spectrometry (LC-MS/MS) analyses. The formed metabolites of CYP probe cocktail (acetaminophen, 4′-hydroxydiclofenac, 4′-hydroxy-S-mephenytoin, 1′-hydroxybufuralol, and 1′-hydroxymidazolam, respectively) were quantified with the LC-MS/MS (LC-20A (Shimadzu, Kyoto, Japan)/SCIEX Triple Quad 5500+ (AB Sciex LLC, Framingham, MA, USA)).

### 2.5. Gene Expression Analysis Using the Focused Microarray Assay

Genopal^®^ focused DNA microarray (Mitsubishi Chemical Co., Ltd., Tokyo, Japan) for hepatocyte evaluation was used for an exhaustive gene expression analysis [45,46]. These chips contain probes for 183 liver-related genes. The isolated RNA (400 ng) was amplified and biotin-labeled using the MessageAmp™ II-Biotin Enhanced aRNA Amplification kit (Applied Biosystems, Foster City, CA, USA) according to the manufacturer’s instructions. The biotinylated, amplified RNA (aRNA, 5μg) was hybridized with the DNA probe on the focused microarray. The DNA microarray was labeled with streptavidin-Cy5 (GE Healthcare Bio-Science KK, Tokyo, Japan). Hybridization signals were acquired using a DNA microarray reader with multibeam excitation technology (Yokogawa Electric Co., Tokyo, Japan).

### 2.6. Data Analysis

The signal from each sample was background corrected and normalized using the median of five control gene signals. Log2 (signal/average signal of all samples) values were used for the PCA and hierarchical clustering. PCA was performed using MetaboAnalyst 4.0 [47]. Hierarchical clustering was performed by calculating Pearson correlation distances in Multiple Experiment Viewer [48]. We selected branches comprising genes whose loading scores in PCA tended to be similar and identified enriched characteristics in these branches. A chi-squared test was performed to determine the statistical significance of the enriched characteristics.

## 3. Results

### 3.1. Comparing the CYP Gene Expression and Activities of Cell Source Candidates for Liver-MPS and CryoHeps

**Figure 1:**
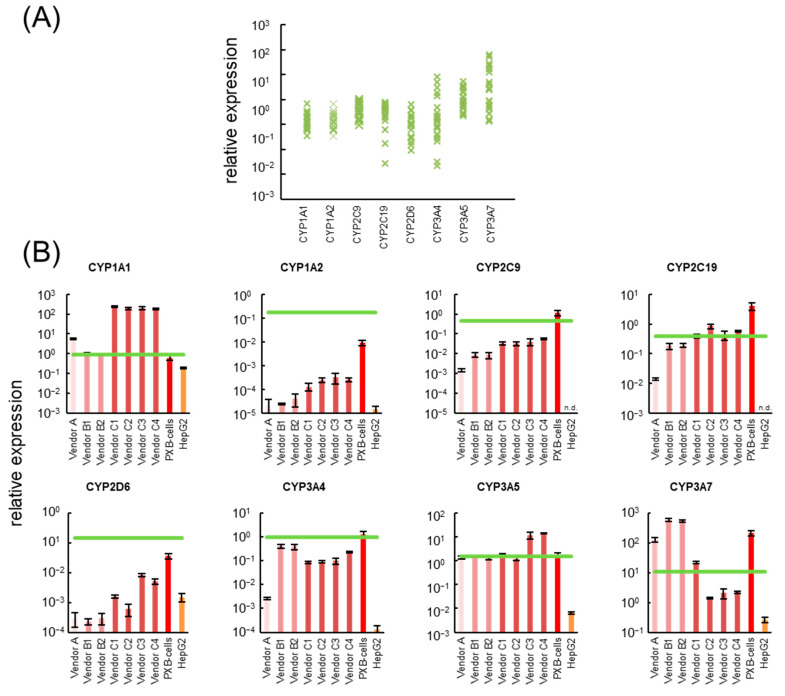
Comparison of the gene expression levels of cytochrome P450 (*CYP*) in cell source candidates for the liver-MPS (hepatocyte-like cells derived from human induced pluripotent stem cells, PXB-cells, and HepG2 cells) with the minimum and maximum values of the expression in 22 lots of cryopreserved human hepatocytes (cryoheps). (**A**) Distribution of *CYPs* expression levels in 22 lots of cryoheps. (**B**) The bars show the gene expression levels of *CYPs* in cell source candidates for the liver-MPS. The green line shows the average value of the expression in 22 lots of cryoheps. Pooled RNA from human liver was used for the standard curve, and the expression level was set as one. The relative expression level was calculated using the equation of the line for the standard curve. *n* = 3. n.d, not detected.

**Figure 2:**
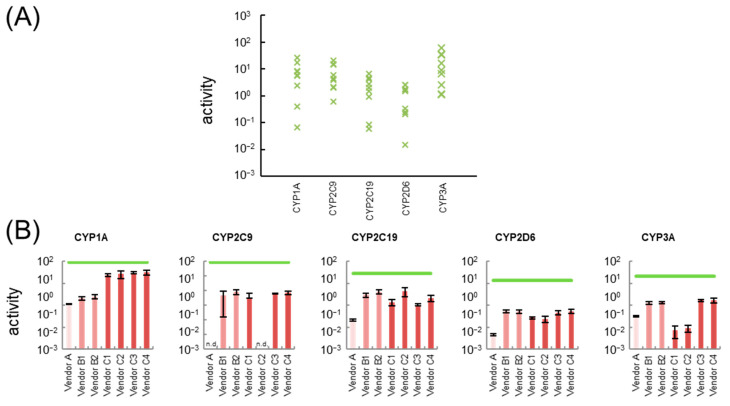
Comparison of the activity levels of cytochrome P450 (CYP) in hepatocyte-like cells derived from human induced pluripotent stem cells (hiPSC-Heps) with the minimum and maximum values of the expression in 8 lots of cryopreserved human hepatocytes (cryoheps). (**A**) Distribution of activity levels of CYPs in 8 lots of cryoheps. (**B**) The bars show the activity level of CYPs in hiPSC-Heps. The green line shows the average value of activity in 8 lots of cryoheps. *n* = 3. n.d, not detected.

Drugs taken up by the hepatocytes are first catalyzed by CYPs. Therefore, CYP gene expression is an important index for the cell-based drug safety test. The gene expression levels of the major *CYP* subfamilies (*CYP1A2*, *CYP2C9*, *CYP2C19*, *CYP2D6*, *CYP3A4*) and other *CYP* subfamilies (*CYP1A1*, *CYP3A5*, *CYP3A7*) of cell source candidates for liver-MPS (hiPSC-Heps, PXB-cells, and HepG2 cells) were compared with 22 lots of cryoheps (Figure 1 and Figure S1). In addition, the activities of the major CYPs of hiPSC-Heps were compared with 8 lots of cryoheps (Figure 2 and Figure S2). Distribution of expression levels and activity levels of CYPs in 22 lots or 8 lots of cryoheps were shown in Figure 1A and Figure 2A. To compare cells under optimal conditions throughout this study, hiPSC-Heps, PXB-cells, and cryoheps were cultured according to the protocol recommended by the vendors and HepG2 cells were cultured in the most common conditions.

In hiPSC-Heps from vendor A, the expression levels of *CYP1A1* and *CYP3A5* were the same as those of cryoheps. Meanwhile, the expression levels of *CYP1A2*, *CYP2C9*, *CYP2C19*, *CYP2D6*, and *CYP3A4*, which are major *CYP* subfamilies, were one to four orders of magnitude lower than those average values in cryoheps. Lastly, the expression levels of *CYP3A7*, known as the primary fetal *CYP3A* enzyme, were one orders of magnitude higher than those of cryoheps. The activity levels of CYP2C9 was not detected, and those of other CYPs were two orders of magnitude lower than average value of cryoheps.

hiPSC-Heps from vendors B1 and B2 were derived from the same donor but different production lots. CYP activity and expression patterns were the same in both cell lots. In hiPSC-Heps from vendor B, the expression levels of *CYP2C19* and *CYP3A4*, which are major *CYP* subfamilies, were the same as those of cryoheps. In addition, the activity levels of CYP2C19 and CYP3A were within difference among lots of cryoheps, but 7- to 15-fold lower than average values. These suggested that CYP2C19 and CYP3A metabolic ability in hiPSC-Heps from vendors B1 and B2 are lower than those in the average lot of cryoheps and higher than those in the worst lot. Meanwhile, the expression levels of *CYP1A2*, *CYP2C9*, and *CYP2D6* and the activity levels of CYP1A, CYP2C9, and CYP2D6 were one to four orders of magnitude lower than average values of cryoheps. Lastly, the expression levels of *CYP3A7*, known as the primary fetal CYP3A enzyme, were one orders of magnitude higher than those of cryoheps.

hiPSC-Heps from vendors C1, C2, and C3 were derived from different donors. hiPSC-Heps from vendors C3 and C4 were derived from the same donor but different production lots. In hiPSC-Heps from vendor C, the expression levels of *CYP2C19*, which is major *CYP* subfamilies, were the same as that of cryoheps. The activity levels of CYP2C19 was within difference among lots of cryoheps, but 6- to 28-fold lower than those average value. These suggested that CYP2C19 metabolic ability in hiPSC-Heps are lower than those in the average lot of cryoheps and higher than those in the worst lot. The activity levels of CYP1A were one-third of average value in cryoheps. Meanwhile, the expression levels of *CYP1A2*, known as the main hepatic CYP1A, were four orders of magnitude lower than average value of cryoheps. Lastly, the expression levels of *CYP1A1*, known as the intestinal CYP1A, were two orders of magnitude higher than average value of cryoheps. These suggested that CYP1A1 is the main contributor to *CYP1A* activity. In hiPSC-Heps from vendors C3 and C4, CYP activity and expression patterns were the same. These activity levels of *CYP3A* were one order of magnitude higher than those in hiPSC-Heps from vendors C1 and C2. Meanwhile, the expression levels of *CYP3A4* were the same as those in hiPSC-Heps from vendors C1 and C2. Lastly, the expression levels of *CYP3A5* were one order of magnitude higher than those in hiPSC-Heps from vendors C1 and C2. These suggested that CYP3A5 is the main contributor of CYP3A activity in hiPSC-Heps from vendor C3 and C4.

In PXB-cells, the expression levels of *CYP2C9*, *CYP2C19*, and *CYP3A4*, which are major CYP subfamilies, were similar or higher than those of cryoheps. In addition, *CYP2D6* expression levels of were within difference among lots, but four-fold lower than average value of cryoheps. Meanwhile, the expression level of *CYP1A2* were one orders of magnitude lower than average value of cryoheps. Lastly, the expression levels of *CYP3A7*, known as the primary fetal CYP3A enzyme, were one orders of magnitude higher than average value of cryoheps.

In HepG2 cells, the expression levels of *CYP1A2*, *CYP2C9*, *CYP2C19*, *CYP2D6*, and *CYP3A4* were not detected or one to five orders of magnitude lower than average values of cryoheps.

### 3.2. Performance Evaluation of the Focused Microaasay

**Figure 3:**
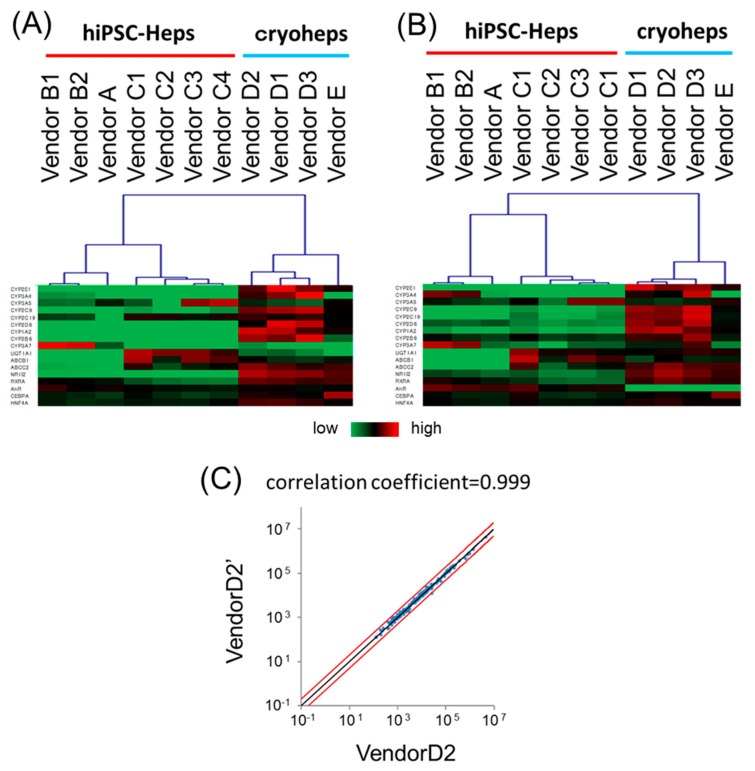
Reliability evaluation of the expression data from the Focused microarray assay. Expression data of 17 drug metabolism genes from the Focused microarray assay were compared with those from a qPCR. (**A**) Heatmap pattern and hierarchical clusters based on expression data from the Focused microarray assay. (**B**) Heatmap pattern and hierarchical clusters based on expression data from the qPCR. The reproducibility of the Focused microarray assay was evaluated. (**C**) Scatter plot of the gene expression data from the Focused microarray assay using cryopreserved human hepatocytes from the same donor. The black line exhibits an equal-fold change, and the red line exhibits a two-fold change.

The cells were observed for differences in *CYP* expression and activity. Thus, the analysis was expanded to include general hepatic characteristics by using a focused microarray. The focused microarray was customized to measure the expression of 183 liver-related genes in one process. Gene probes related to the metabolism of drugs, lipids, carbohydrates, and amino acids and components secreted into the blood are set on the chip to evaluate general liver functions. Furthermore, gene probes related to the makers of immature hepatocytes, non-parenchymal cells, and cancer cells are set on the chip to evaluate cell state. Raw data from the focused microarray assay are shown in Table S2. To evaluate the chip’s performance, we compared the gene expression data from the focused microarray assay with those from a quantitative PCR (qPCR) (Figure 3A,B). Based on the expression data of 17 genes, the focused microarray assay showed a similar heatmap pattern and clusters to those of the qPCR. These suggested that the expression data from the focused microarray assay correlated with those from the qPCR.

Second, the reproducibility of the measurements in the focused microarray was evaluated. The expression of two RNAs from the same donor were independently measured using several chips, and the corresponding results were compared. In a scatter plot based on the expression data of the two RNAs, the plots of almost all genes were located on the line y = x, and the correlation coefficient was 0.999, suggesting that the focused microarray assay is extremely reproducible (Figure 3C). Therefore, the focused microarray will be a useful tool for hepatocyte characterization.

### 3.3. Characteristic Comparison between Cells Based on the Expression Data from the Focused Microarray Assay

**Figure 4:**
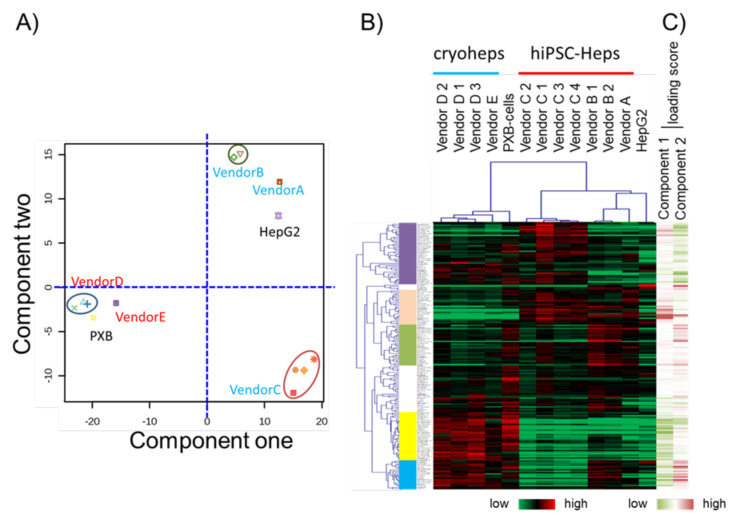
Global characterization of the cell types based on the gene expression data from the Focused microarray assay. (**A**) Principal component analysis (PCA) object. The horizontal axis represents the loading scores for PC1, and the longitudinal axis represents the loading scores for PC2. (**B**) Heatmap patterns and hierarchical clusters. (**C**) The lines show the loading scores for PC 1 and 2. Red lines exhibit plus scores, and green lines exhibit minus scores. Branches comprising genes that tend to have the same loading score are exhibited by color. Orange branches comprise genes with a high loading score for PC1. Yellow branches comprise genes with a low loading score for PC1. Green branches comprise genes with a high loading score for PC2. Purple branches comprise genes with a low loading score for PC2. Blue branches comprise genes with a low loading score for PC1 and a high loading score for PC2.

The aforementioned results suggested that the focused microarray assay is a reliable method for gene expression measurement. Thus, using the expression data from the focused microarray assay, we compared the characteristics of the cell types through PCA and hierarchical clustering. For comparison with alternative cells, cryoheps from wendor D, which tends to have high *CYPs* expression, and vendor E, which tends to have low *CYPs* expression, were used for analysis. In the PCA score plot, PC1 grouped cryoheps and PXB-cells, against hiPSC-Heps and HepG2 cells (Figure 4A). Meanwhile, PC2 grouped hiPSC-Heps from vendors A and B against hiPSC-Heps from vendor C. The hierarchical clustering of the samples generated a cluster including cryoheps and PXB-cells and a cluster including hiPSC-Heps and HepG2 cells (Figure 4B). These suggested that PXB-cells and cryoheps have similar characteristics. Likewise, hiPSC-Heps and HepG2 cells have similar characteristics. In addition, PC2 suggested that hiPSC-Heps from vendors A and B and hiPSC-Heps from vendor C have different characteristics.

### 3.4. Gene Classification by Expression Pattern and Biological Interpretation by Enrichment Analysis

**Figure 5:**
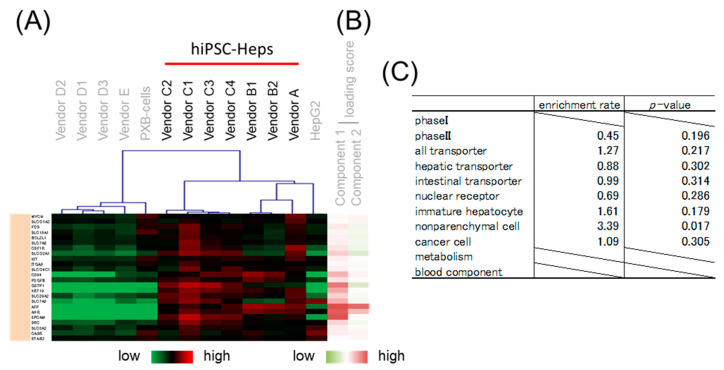
Enrichment analysis of the orange branch. (**A**) Heatmap patterns. (**B**) PC 1 and 2 scores. Red lines exhibit plus scores, and green lines exhibit minus scores. (**C**) Enrichment rates and *p*-values from the chi-squared test of different gene categories.

**Figure 6:**
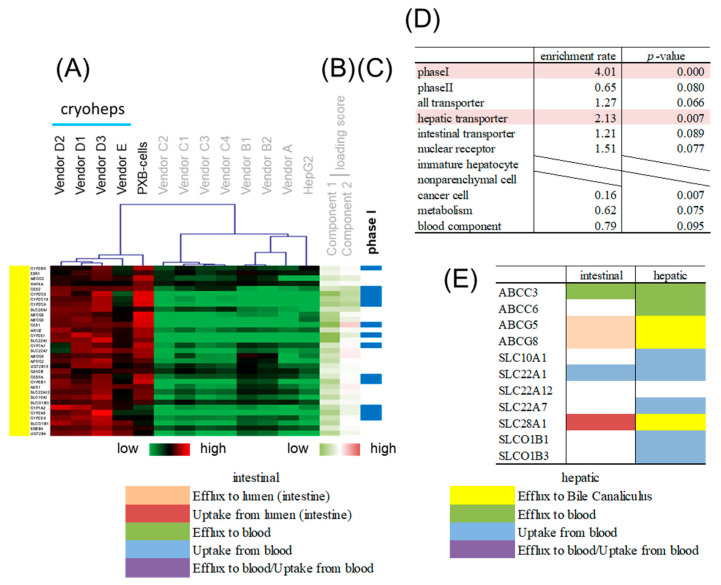
Enrichment analysis of the yellow branch. (**A**) Heatmap patterns. (**B**) PC 1 and 2 scores. Red lines exhibit plus scores, and green lines exhibit minus scores. (**C**) The blue lines exhibit genes related to drug metabolism phase I enzymes. (**D**) Enrichment rates and *p*-values from the chi-squared test of different gene categories. The highlighted categories are significantly enriched. (**E**) Transporter genes. Hepatic and intestinal transporter genes are classified according to function, as shown by color.

**Figure 7:**
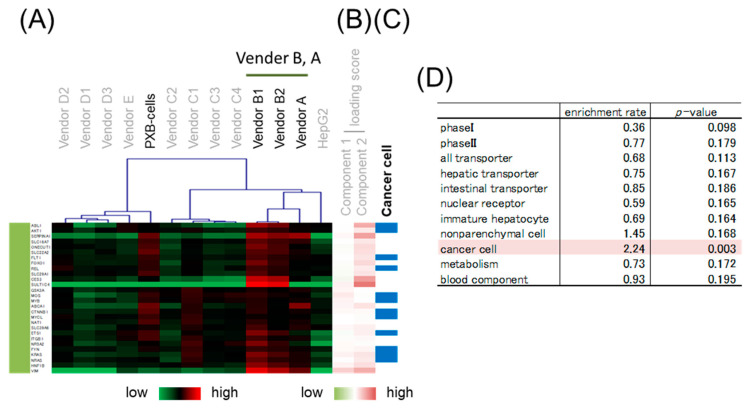
Enrichment analysis of the green branch. (**A**) Heatmap patterns. (**B**) PC 1 and 2 scores. Red lines exhibit plus scores, and green lines exhibit minus scores. (**C**) The blue lines exhibit genes related to cancer cells. (**D**) Enrichment rates and *p*-values from the chi-squared test of different gene categories. The highlighted categories are significantly enriched.

**Figure 8:**
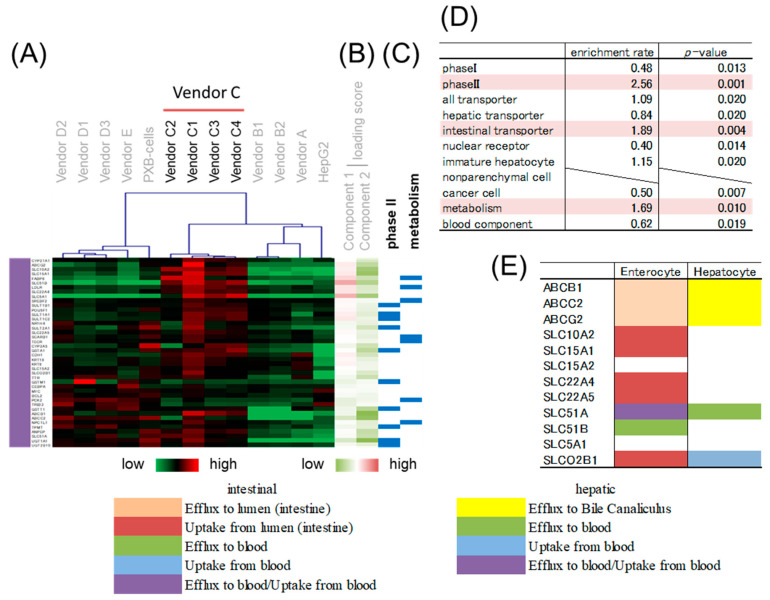
Enrichment analysis of the purple branch. (**A**) Heatmap patterns. (**B**) PC 1 and 2 scores. Red lines exhibit plus scores, and green lines exhibit minus scores. (**C**) The blue lines exhibit genes related to drug metabolism phase II enzymes. (**D**) Enrichment rates and *p*-values from the chi-squared test of different gene categories. The highlighted categories are significantly enriched. (**E**) Transporter genes. Hepatic and intestinal transporter genes are classified according to function, as shown by color.

**Figure 9:**
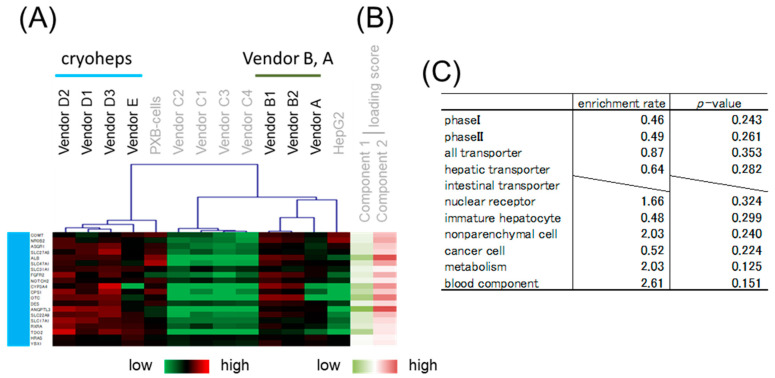
Enrichment analysis of the blue branch. (**A**) Heatmap patterns. (**B**) PC 1 and 2 scores. Red lines exhibit plus scores, and green lines exhibit minus scores. (**C**) Enrichment rates and *p*-values from the chi-squared test of different gene categories.

Hierarchical clustering was performed to classify genes whose expression patterns are similar. We selected branches collecting genes that tend to have the same loading score in PCA (Figure 4B,C). Among the samples, five branches were found to have expression levels that characteristically changed as shown by color. Enrichment analysis was performed to evaluate the characteristics of genes enriched in branches of each color. The genes on the focused microarray were classified as shown in Table S3, and the significance of the enrichment was evaluated through a chi-squared test. The level of significance was set at *p* < 0.01 and enrichment fold > 1.5. Genes with a high loading score for PC1 were included in an orange branch, and their expression levels were high in hiPSC-Heps and HepG2 cells. In this branch, non-parenchymal cell markers and immature hepatocyte markers were enriched by more than 1.5-fold, albeit nonsignificantly (Figure 5). Genes with a low loading score for PC1 were included in a yellow branch, and their expression levels were high in cryohepatocytes and PXB-cells. In this branch, genes related to drug metabolism phase I enzymes and hepatic transporters were significantly enriched (Figure 6). Additionally, 10 of the 11 genes related to transporters were hepatic transporters (Figure 6E). Genes with a high loading score for PC2 were included in a green branch, and their expression levels were high in PXB-cells and hiPSC-Heps from vendors A and B. In this branch, genes related to cancer cells were significantly enriched (Figure 7). Genes with a low loading score for PC2 were included in a purple branch, and their expression levels were high in hiPSC-Heps from vendor C. In this branch, genes related to drug metabolism phase II enzymes, intestinal transporters, and metabolism were significantly enriched (Figure 8). Additionally, 10 of the 12 genes related to transporters were intestinal transporters (Figure 8E). Genes with a low loading score for PC1 and a high loading score for PC2 were included in a blue branch, and their expression levels were high in cryohepatocytes, PXB-cells, and hiPSC-Heps from vendors A and B. In this branch, genes related to nuclear receptors, non-parenchymal cell markers, metabolism, and blood components were enriched by more than 1.5-fold, albeit nonsignificantly (Figure 9).

### 3.5. Gene Expression Analysis of Intestinal Markers

**Figure 10:**
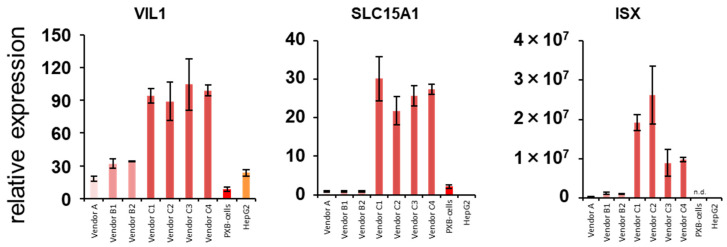
Gene expression levels of intestinal markers in human hepatocytes. The gene expression levels of intestinal markers in cell source candidates for the liver-MPS (hiPSC-Heps, PXB-cells, and HepG2 cells) were measured by qPCR. Pooled RNA from human liver was used for the standard curve, and the expression level was set as one. The relative expression level was calculated using the equation of the line for the standard curve. *n* = 3. n.d: not detected.

In the branch shown in Figure 8, genes related to intestinal transporters were significantly enriched, and their expression levels were high in hiPSC-Heps from vendor C. Additionally, the expression level of *CYP1A1*, known as the intestinal *CYP1A*, in these cells was much higher than that in other hepatocytes. These results suggested the possibility of finding the intestinal characteristics in hiPSC-Heps from vendor C. Therefore, the expression levels of *VIL1*, *ISX*, and *SLC15A1*, which are intestinal markers, were measured by qPCR [49]. The expression of any intestinal marker in hiPSC-Heps from vendor C was higher than that in other hepatocytes (Figure 10), suggesting the possibility of mixing the intestinal characteristics of enterocytes in them.

## 4. Discussion

Animal experiments have been conducted to evaluate pharmacokinetics, toxicity, and usefulness in drug development. However, these experiments involve species different from humans and are limited by animal welfare restrictions. MPS comprise culture techniques for reconstructing the specific functions of human organs or tissues in a limited space, creating miniaturized human test systems. These have been actively developed in recent years [2,3,4,5,6,7,8,9,10,11,12,13,14,15]. In MPS development, the choice of cell source to mount on the MPS is as important as the design of the device. In drug development, to confirm efficacy, and evaluate the occurrence of side effects and adverse events, cells with liver-like functions are required. In addition, efficacy and safety tests for candidate compounds are repeated over time. Therefore, we considered that high liver function and a sustainable supply of cells with the same performance are important selection criteria for the cell source to be mounted on the liver-MPS [9]. Cryoheps are regarded as gold standard hepatocytes; however, cryoheps has different liver functions among donors. As the truth, in the cryoheps used in this study, the *CYPs* expression level and activity differed among donors by up to one to three orders of magnitude. Likewise, careful selection of cryoheps not only by the cell functions but also by cell plateability may be required for the optimal cell source to mount on the liver-MPS. Pooled donor lots might be good alternatives to overcome difference among donors, however the caution may take into account that the *CYPs* expression level differed by up to an order of magnitude. Therefore, cryoheps may not be the optimal cell source to mount on the liver-MPS. Three types of hepatocytes examined in this study, namely PXB-cells, hiPSC cells, and HepG2 cells, can be sustainably supplied with the same characteristics. Thus, repeated tests for long periods may be possible with these three types of hepatocytes. In this regard, these cells are suitable as a cell source for MPS. The liver functions that are equivalent to cryoheps are also required in order to utilize these cells for the liver-MPS. Therefore, the liver-like characteristics of these cells were compared with those of cryoheps. The PCA analysis and hierarchical clustering of the samples showed that the liver-like characteristics of PXB-cells were extremely similar to those of cryoheps, suggesting that hepatocytes derived from liver-humanized mice cells are promising alternatives to cryoheps. Additionally, their drug metabolism ability required for ADME prediction was mostly equivalent to that of cryoheps. Yamasaki et al. reported that all CYPs activities (CYP1A, CYP2C9, CYP2C19, CYP2D6, CYP2E1, CYP3A) measured in PXB-cells is comparable to that of cryopreserved human hepatocytes [50]. Thus, these may be the most suitable as alternative cell sources to mount on the liver-MPS for safety evaluation, including ADME of drugs. These suggested that drug metabolism, toxicity, and usefulness can be determined by repetitive evaluation over long periods with stable functionality if hepatocytes derived from liver-humanized mice cells are mounted on the liver-MPS. However, the expression level of *CYP1A2* in PXB-cells was higher than that of hiPSC-Heps but one order of magnitude lower than average value of cryoheps. Furthermore, the expression level of *CYP3A7*, known as the primary fetal CYP3A enzyme, and cancer cell-related genes tended to be high in PXB-cells. These suggested that the cells may be immature. The expression of *CYP1A2* may be improved by maturing PXB-cells.

The PCA analysis and hierarchical clustering of the samples based on the data from the focused microarray assay showed that the characteristics of hiPSC-Heps were closer to those of HepG2 than those of cryoheps. Additionally, the enrichment analysis revealed that the expression levels of drug metabolism phase I enzymes and hepatic transporters were lower in hiPSC-Heps than in cryoheps. These suggested that improving the expression of drug metabolism phase I enzymes and hepatic transporters is required when using hiPSC-Heps as alternatives to cryoheps. Likewise, Kratochwil et al. reported that the metabolic activity in hiPSC-Heps and HepG2 cells was more than tenfold lower [51]. However, *CYPs* expression levels in hiPSC-Heps were higher than that in HepG2 cells, and some expression levels were same with those average values in cryoheps. The metabolic capacity of CYPs in hiPSC-Heps can be evaluated by measuring metabolites through LC-MS/MS, and some CYPs activity levels were lower than average values in cryoheps, but higher than lowest value. These suggested that some metabolic ability in hiPSC-Heps are lower than those in the average lot of cryoheps but higher than those in the worst lot. In addition, the enrichment analysis suggested that some hepatic functions of hiPSC-Heps are at the same level as those of cryoheps. For example, the expression trends of genes related to blood components and basal metabolism in hiPSC-Heps from vendor B and those related to drug metabolism phase II enzymes and basal metabolism in hiPSC-Heps from vendor C were similar to those of cryoheps. Semi-permanently supplying hiPSC-Heps from the same donor can be achieved by proliferating human iPS cells and differentiating these into hepatocytes. In this study, the expression levels and activities of CYPs among hiPSC-Heps from the same donor but different production lots (vendors B1 and B2, vendors C3 and C4) were at the same level. In addition, the hierarchical clustering of the samples baed on the data from the focused microarray assay showed that hiPSC-Heps from the same donor but different production lots were adjacent to each other. These suggested that the method for establishing hiPSC-Heps is extremely reproducible. Overall, hiPSC-Heps allows for a repetitive evaluation of drug safety over long periods with stable functionality. Furthermore, these can create cells for disease models [52,53], which is useful for drug development and safety evaluation in special cases. Additionally, we confirmed that the bile canaliculi, which were required for a cholestasis toxicity test and biliary efflux evaluation, were formed in hiPSC-Heps from one vendor [54]. In conclusion, hiPSC-Heps has advantageous characteristics that are not found in cryoheps, and may be unique cell source candidates for liver-MPS although improvement of the expression of *CYPs* and hepatic transporters is required.

HepG2 cells are easier to use than the other cells because they can proliferate. At present, these are generally used as alternative cell sources to cryoheps. However, the expression levels of liver-related genes related were predominantly lower than those of cryoheps. Therefore, although HepG2 cells would be suitable for basic research for liver-MPS at the laboratory level, but not for safety evaluation including ADME in drug development.

In this study, the concomitance of intestinal characteristics of hepatocyte-like cell evaluation were raised as points of consideration. In hiPSC-Heps from vendor C, the expression level of *CYP1A1*, which is an intestinal CYP, was remarkably high compared to that of the other hepatocytes. Likewise, intestinal transporter genes were enriched in branches whose expression levels were high in these cells. Additionally, intestinal *CES2* expression levels/hepatic *CES1* expression levels in hiPSC-Heps from vendor C was high compared to that of the other hepatocytes. Therefore, when the expression levels of intestinal markers were examined, their expression level tended to be predominantly high in hiPSC-Heps from vendor C. In addition, enterocytes and hepatocytes are differentiated from the embryonic endoderm [55]. These suggested the possibility of having intestinal characteristics in hiPSC-Heps from vendor C, due to insufficient differentiation from the embryonic endoderm to hepatocytes. Such cells should be careful evaluation as candidate cells for liver-MPS. Cells differentiated from iPS and embryonic stem cells have been evaluated using genes related to target tissues as indicators up to the present time. However, the possibility of finding characteristics of other tissues must be considered when evaluating hepatocytes derived from iPS and embryonic stem cells, and markers of tissues that differentiate from the mesoderm should be included as indices when evaluating these hepatocytes.

Multiple CYPs are involved in the reaction of the substrate used to measure CYP1A and CYP3A activities. CYP1A2 and CYP3A4 is the main contributor of CYP1A and CYP3A activity in cryohep. In this study, the *CYPs* expression data suggested that a type of CYP mainly contributed in CYP1A and CYP3A activities of some hiPSC-Heps is different from that of cryoheps. Therefore, we think that the evaluation of drug metabolism in various types of hepatocytes should include both gene expression and activity.

We could evaluate the liver-like characteristics and demonstrate the consideration points as an alternative cell source candidate for liver-MPS. However, the ability to form bile canaliculi, which is required for a cholestasis toxicity test and biliary efflux evaluation, cannot be evaluated with the analysis methods in this study. Biliary efflux evaluation is important for ADME predictions [50,56,57]. Identifying indicator genes for bile canaliculi forming ability and including them in the focused microarray may enable the selection of cells more suitable for ADME predictions. The number of lots for CYPs activity data was less compared to those for expression data. In addition, many lots for CYPs activity data tended to have high *CYPs* expression levels. Therefore, the lowest value of CYP activity level in cryoheps was up to two orders of magnitude lower than the average value, although it was fully evaluable by LC-MS/MS. Hence, in order to know more accurate CYPs activity distribution between lots, the activity must be measured in many lots. We are planning to measure CYPs activity and expression level in more hepatocytes to contribute determine baseline activity values for cells used in safety studies, including ADME. Likewise, evaluation about drug-metabolizing enzymes, including UGT, CYP1A3, CYP1A4, CYP1A6, CYP1A9, CYP2B7 and CYP2B15, is a future task.

## 5. Conclusions

In this study, we performed a characteristic analysis of three types of hepatocytes and evaluated their potential as alternative cell sources to cryoheps. Based on these results, consideration points for using them as the cell source for the liver-MPS could be indicated. In addition, we suggested points for improving three types of cells as hepatocytes with liver-like characteristics. Findings in this study will guide the selection of more suitable cells for liver-MPS and contribute to the repetitive utilization of a robust in vitro hepatic assay system over long periods with stable functionality. As a result, this study will lead to the promotion of the utilization of MPS in drug development.

## Data Availability

The datasets used and/or analyzed during the current study are available from the corresponding author on reasonable request.

## Supplementary Materials

The following supporting information can be downloaded at: https://www.mdpi.com/article/10.3390/pharmaceutics15010055/s1, Figure S1. Gene expression levels of cytochrome P450 (CYP), which plays a major role in drug metabolism in hepatocyte-like cells derived from human induced pluripotent stem cells (hiPSC-Heps), PXB-cells, HepG2 cells, and cryopreserved hepatocytes (cryoheps). Pooled RNA from human liver was used for the standard curve, and the expression level was set as one. *n* = 3. Figure S2. Activity levels of cytochrome P450 (CYP), which plays a major role in drug metabolism in hepatocyte-like cells derived from human induced pluripotent stem cells and cryopreserved hepatocytes. *n* = 3. Table S1. Information on human cryopreserved hepatocytes used in this study. Table S2. Raw data of focused microarray. Table S3. Gene list of focused microarray.
