# Supplementary material for: Consideration of Commercially Available Hepatocytes as Cell Sources for Liver-Microphysiological Systems by Comparing Liver Characteristics
Source: Pharmaceutics. 2022 Dec 24;15(1):55. doi: 10.3390/pharmaceutics15010055 (PMC9867117; doi:10.3390/pharmaceutics15010055)
Supplement: Supplementary file 1 [file pharmaceutics-15-00055-s001.zip › pharmaceutics-2088143-supplementary/Supplemtary figure.pdf]

		relative expressions							
		CYP1A1	CYP1A2	CYP2C9	CYP2C19	CYP2D6	CYP3A4	CYP3A5	CYP3A7
hiPSC-Heps	Vendor A	5.6	0.000021	0.0015	0.014	0.00030	0.0026	1.4	130
	Vendor B 1	1.0	0.000024	0.0089	0.18	0.00023	0.42	1.5	590
	Vendor B 2	0.88	0.000040	0.0086	0.19	0.00030	0.38	1.3	550
	Vendor C 1	230	0.00013	0.033	0.40	0.0016	0.087	1.7	22
	Vendor C 2	190	0.00025	0.033	0.84	0.00061	0.091	1.2	1.4
	Vendor C 3	200	0.00032	0.040	0.43	0.0084	0.10	12	2.1
	Vendor C 4	180	0.00026	0.055	0.58	0.0051	0.23	14	2.2
PXB-cells	PXB-cell s	0.63	0.0092	1.2	3.9	0.037	1.3	1.7	210
HepG2	HepG2	0.20	0.000014	not detected	not detected	0.0015	0.00013	0.0066	0.27
cryoheps	Vendor D 1	0.26	0.35	0.94	0.43	0.36	4.9	1.3	0.93
	Vendor D 2	0.62	0.35	0.72	0.71	0.10	3.1	2.0	2.8
	Vendor D 3	0.11	0.11	1.1	0.65	0.24	8.4	0.93	51
	Vendor E 1	0.43	0.066	0.15	0.38	0.020	0.072	2.0	4.2
	Vendor E 2	0.20	0.16	0.37	0.017	0.18	0.18	0.78	28
	Vendor E 3	0.53	0.035	0.10	0.0028	0.028	0.0032	0.60	0.42
	Vendor E 4	0.27	0.066	0.13	0.6138	0.034	0.0212	0.67	0.53
	Vendor E pooled 1	0.79	0.089	0.25	0.064	0.10	0.087	3.2	13
	Vendor E pooled 2	0.44	0.23	0.34	0.39	0.18	0.30	2.2	23
	Vendor F 1	3.7	0.69	0.78	0.19	0.11	0.21	0.67	0.16
	Vendor F 2	1.3	0.065	0.38	0.26	0.029	0.028	0.47	0.14
	Vendor F 3	2.7	0.27	0.35	0.21	0.087	0.16	0.93	0.28
	Vendor F 4	2.8	0.19	0.29	0.56	0.030	0.19	0.23	0.28
	Vendor F 5	2.3	0.29	0.68	0.51	0.10	0.13	0.25	0.16
	Vendor F 6	0.87	0.062	0.67	0.38	0.087	0.0023	1.2	0.63
	Vendor G 1	0.20	0.071	0.16	0.60	0.015	0.040	0.30	2.5
	Vendor G 2	0.31	0.21	0.27	0.49	0.061	0.22	5.3	5.2
	Vendor G 3	0.42	0.14	0.18	0.51	0.0091	0.076	2.1	2.8
	Vendor G pooled 1	1.72	0.13	0.17	0.77	0.12	0.039	3.8	0.9
	Vendor H 1	0.11	0.056	0.44	0.15	0.23	0.48	0.53	17
	Vendor H 2	0.026	0.13	0.59	0.35	0.64	1.4	0.28	27
	Vendor H 3	0.35	0.084	0.69	0.47	0.40	1.8	2.9	63

Figure S1. Gene expression levels of cytochrome P450 (*CYP*), which plays a major role in drug metabolism in hepatocyte-like cells derived from human induced pluripotent stem cells (hiPSC-Heps), PXB-cells, HepG2 cells, and cryopreserved hepatocytes (cryoheps). Pooled RNA from human liver was used for the standard curve, and the expression level was set as one. n = 3.

		activity (pmol / million cells / min)				
		CYP1A (Acetaminophen)	CYP2C9 (4'-OH dicrofenac)	CYP2C19 (4'-OH S-mephenytoin)	CYP2D6 (1'-OH bufuralol)	CYP3A (1'-OH midazolam)
hiPSC-Heps	Vendor A	0.11	not detected	0.022	0.0045	0.32
	Vendor B 1	0.21	0.048	0.30	0.054	1.2
	Vendor B 2	0.24	0.085	0.42	0.051	1.3
	Vendor C 1	2.3	0.031	0.14	0.027	0.071
	Vendor C 2	2.7	not detected	0.44	0.024	0.090
	Vendor C 3	3.0	0.063	0.10	0.046	1.7
	Vendor C 4	3.1	0.074	0.21	0.054	1.7
cyoheps	Vendor D 1	8.0	3.9	4.2	2.6	6.8
	Vendor D 2	5.7	2.0	2.0	0.34	2.6
	Vendor D 3	8.5	4.5	6.7	2.5	16
	Vendor E 1	0.41	2.1	0.059	0.21	1.1
	Vendor E pooled 2	2.4	6.0	0.93	0.26	9.2
	Vendor H 1	26	22	1.5	1.7	34
	Vendor H 2	18	16	2.7	1.5	35
	Vendor H 3	6.0	14.9	4.6	1.8	60

Figure S2. Activity levels of cytochrome P450 (CYP), which plays a major role in drug metabolism in hepatocyte-like cells derived from human induced pluripotent stem cells and cryopreserved hepatocytes. *n* = 3.
